# Neuropathic Pain in Children with Sickle Cell Disease: The Hidden Side of the Vaso-Occlusive Crisis

**DOI:** 10.3390/children8020084

**Published:** 2021-01-26

**Authors:** Jeanne Sigalla, Nathalie Duparc Alegria, Enora Le Roux, Artemis Toumazi, Anne-Françoise Thiollier, Laurent Holvoet, Malika Benkerrou, Sophie Dugue, Berengere Koehl

**Affiliations:** 1 Pain Management Unit, Hôpital Robert Debré, APHP, F-75019 Paris, France; jeanne.sigalla@aphp.fr (J.S.); nathalie.duparc@aphp.fr (N.D.A.); anne.thiollier@aphp.fr (A.-F.T.); 2 Unité d’Épidémiologie Clinique, Inserm, CIC 1426, Hôpital Universitaire Robert Debré, AP-HP, Nord-Université de Paris, F-75019 Paris, France; enora.leroux@aphp.fr (E.L.R.); artemis.toumazi@aphp.fr (A.T.); 3 Université de Paris, ECEVE UMR 1123, Inserm, F-75010 Paris, France; malika.benkerrou@aphp.fr; 4 Department of Hematology, Reference Center of Sickle Cell Disease, Hôpital Universitaire Robert Debré, AP-HP, Nord-Université de Paris, F-75019 Paris, France; laurent.holvoet@aphp.fr; 5 Pain Management Unit, Trousseau Hospital, AP-HP, Sorbonne Université, F-75012 Paris, France; sophie.dugue@aphp.fr; 6 INSERM UMRS 1134, Red Blood Cell Pathophysiology, F-75015 Paris, France

**Keywords:** sickle cell disease, neuropathic pain, vaso-occlusive crisis, pediatrics, DN-4

## Abstract

The majority of hospitalizations of patients with sickle cell disease (SCD) are related to painful vaso-occlusive crises (VOCs). Although the pain of VOC is classically nociceptive, neuropathic pain (NP) has also been demonstrated in SCD patients. The aim of our study is to specify the prevalence of NP during VOCs in SCD children using a dedicated scale and to measure its characteristics. We performed a prospective study that included SCD children hospitalized for an acute VOC. The presence of NP was sought with the DN4 scale on the second and fourth days of hospitalization. A total of 54 SCD children were included in the study. Overall, 41% of the patients (*n* = 22) experienced neuropathic pain during the VOC, mostly at an early stage (Day 2). The median age, the sex ratio, the location of the pain, and the morphine consumption were similar for patients with and without NP. Our study shows that neuropathic pain is very common during VOCs in SCD children. The absence of identified risk factors should prompt us to be vigilant regardless of the patient’s age, sex, and clinical presentation.

## 1. Introduction

Sickle cell disease (SCD) is a genetic hemoglobinopathy resulting from a unique mutation in the β-globin gene characterized by hemolytic anemia, painful vaso-occlusive crisis (VOC), and progressive organ failure. SCD is the most common hemoglobinopathy worldwide, with approximately 300,000 new cases each year and millions of patients, mainly in Africa, but also in the United States, Europe, and South America [1]. A total of 95% of hospitalizations of patients with SCD are related to acute painful episodes caused by VOCs [2]. Vaso-occlusion is a multifactorial process involving occlusion of small blood vessels by sickled red blood cells (RBCs), polymorphonuclear neutrophils, platelets, and activated endothelial cells. This occlusive process leads to hypoxia and then ischemia of the upstream territories, which is associated with local inflammation [3]. This local inflammation activates the release of the substance P from the nociceptive fibers, generating a nociceptive message and pain by excess nociception [4]. The endothelial and immune systems are also involved, reinforcing the release of pro-algogen molecules. This local inflammation is responsible for an amplification of the nociceptive message and can lead to peripheral hyperalgesia (clinically manifested by a lowering of nociceptive thresholds or allodynia) and can lead to central sensitization. Clinical manifestations of the VOC often start with a prodromal phase of 1–2 days, with pain peaking on day 3 and lasting until day 6 or day 7 before resolving [2]. However, the pattern of the crisis can be very heterogeneous from one patient to another, or even from one episode to another for the same patient, with sometimes sudden and violent crises from the outset. The intensity of the pain is extreme, affecting one or more of the joints, limbs, or back, with sensations of crushed bones and torsion of the limbs described by patients from an early age [5]. The intensity of the pain commonly requires intravenous opioid treatments. Recurrent episodes have a significant impact on quality of life (QOL) [6], so identification, treatment, and prevention of VOCs is crucial for lowering the impact of the disease on the morbidity and the global QOL in these patients. Management of VOCs begins with rapid, effective analgesic treatment adapted to the intensity and the nature of the pain [7]. Although the pain of vaso-occlusive episodes is classically nociceptive, neuropathic pain (NP) has also been demonstrated in SCD patients, both in adults [8] and in children aged 7 years and above, by using quantitative sensory testing (QST) [9]. There is no real consensus on the definition of the criteria for neuropathic pain in children. The International Association for the Study of Pain defines neuropathic pain as “pain initiated or caused by a primary lesion or dysfunction in the nervous system.” The classification proposed by the International Association for the Study of Pain (IASP), either nociceptive or neuropathic pain, leaves out a considerable number of children presenting with symptoms of both; therefore, it is referred to as “mixed pain” by clinicians. As discussed in a recent review, the use of this concept can have therapeutic consequences [10]. The distribution between neuropathic and mixed pain cases in daily practice is around one-third versus two-thirds, as estimated by respondents in the survey of de Leeuw et al. [11]. Trauma and operation are mostly regarded as the first cause of neuropathic and/or mixed pain in children, followed by complex regional pain syndrome, cancer-related pain, and phantom limb pain. Neuropathic pain in SCD has essentially been reported as chronic pain, described as numb, tingling, lancinating, spontaneous, shooting, or paroxysmal pain, and is sometimes associated with a sensation of pins and needles, hyperalgesia, and allodynia [12]. These pains are typically resistant to classical anti-nociceptive treatments, and can be responsible for a high consumption of opioids, which underlines the importance of their specific diagnosis. Nevertheless, NP during VOC remains difficult to specifically diagnose and to differentiate from nociceptive pain, especially in children. The DN4 questionnaire is a clinician-administered questionnaire, which has been validated for adults [13] and is commonly used in children [14], for whom there is a lack of a consensual and validated scale for NP. It is presented in the form of seven questions (illustrated with images in its pediatric form) and three clinical examination items (hypo- or hyperesthesia to the touch and to the puncture and the sign of allodynia). The aim of our study is to evaluate the prevalence of NP during VOC in SCD children using the DN4 scale and to specify its risk factors and characteristics in order to better understand and manage them.

## 2. Materials and Methods

### 2.1. Population and Study Design

SCD patients (Hemoglobin SS, SC, or Sβ^0^) aged 6 to18 years old and hospitalized for an acute VOC were included in a prospective study between September 2018 and December 2019. They were all followed up for their chronic disease in the sickle cell disease center of Robert Debré Hospital in Paris. On the second and fourth days of hospitalization for severe VOC (defined by the need for intravenous morphine treatment), the presence of neuropathic pain was sought with the DN4 scale. Demographic/anamnestic data and prescribed analgesic treatments were collected concomitantly on Day 2 and Day 4.

### 2.2. Diagnosis of Neuropathic Pain

Acute neuropathic pain was diagnosed using the pediatric DN4 scale. The scale consists of seven questions on the sensation of pain (burning, swarming, etc.) illustrated with age-appropriate pictures and three parameters sought via clinical examination (hypo/hyperalgesia, allodynia). If the patients answered “Yes” to four or more questions of the 10 above, they were considered as having neuropathic pain. The DN4 scale was administred either by a specialized nurse from the pediatric pain center of the hospital or by a senior practitioner.

### 2.3. Ethical Consideration

The study was conducted in accordance with the declaration of Helsinki and was approved by the French Ethical Committee (CPP Sud Mediterranée 18.029). The collection of clinical and biological data was registered at the French National Commission for Information Technology and Civil Liberties (CNIL, ref 2139039). The parents or legal guardians of the minor patients were informed and gave their consent for the study.

### 2.4. Data Collection

The data were prospectively collected in a standardized form from medical files and included the following items: demographic data (sex, age, genotype, weight), severity of the chronic disease (frequency of hospitalized VOC > 3 per year, past history of acute chest syndrome), chronic treatment (hydroxyurea, ongoing transfusion program), characteristics of the crisis (location of the pain, intensity of the nociceptive pain quantified by visual analog scale (VAS), Numeric Pain Rating Scale (NPRS), or Face Pain Scale—Revised (FPS-R)), date of the onset of the crisis, DN4 results in detail, and medical treatment of the current VOC (analgesic treatments and their doses, hydration).

### 2.5. Statistical Data

Descriptive statistics were used to characterize patients and to determine the prevalence of NP within the cohort. Qualitative variables were described as numbers (percentage) and quantitative variables as medians (1st–3rd quartiles). To compare the characteristics of patients with and without NP, a Khi2 test was performed on qualitative variables and the Student test (*t*-test) was performed on quantitative parameters. *p*-values < 0.05 were considered statistically significant. All analyses were performed with SAS v9.4 (SAS Institute Inc., Cary, NC, USA).

## 3. Results

### 3.1. Characteristics of the Patients

During the inclusion period, the study was proposed to every SCD patient that was aged 6 to 18 years old and hospitalized for an acute VOC. For logistical reasons, we included only the patients admitted to the hospital between Saturday and Monday to be sure that they could be evaluated on Days 2 and 4, avoiding the weekend days. A total of 100% of the patients proposed for the study agreed to participate. A total of 54 SCD children were then included in the study. The sex ratio (F/M) was 1.7, and the median age was 15.0 years old (Q1:11; Q3:16). A total of 83% of the patients (*n* = 45) had an SS genotype, 6% (*n* = 3) had an SC genotype, and 11% (*n* = 6) had an Sβ° genotype. A total of 78% (*n* = 42) had chronic hydroxyurea treatment. A total of 68.5% (*n* = 37) were considered as having a severe profile of SCD because of a past history of acute chest syndrome and/or the occurrence of at least three hospitalized VOCs in the last 12 months. The demographic and anamnestic data are summarized in Table 1.

### 3.2. Characteristics of the Vaso-Occlusive Crisis

A total of 69% of the patients (*n* = 37) had pain in one or more limbs, while the remaining 31% had abdominal, thoracic, or lumbar pain. On the second day of hospitalization, the average morphine intake was 1.2 mg per kilogram of weight and per day (Q1:0.9; Q3:1.6), with a concomitant prescription of per os (po) paracetamol (15 mg/kg/6 h) and intravenous (iv) hyperhydration (1.5 to 2 L/m^2^/day) for all the patients. A total of 81% (*n* = 44) had a concomitant prescription of iv nefopam (1 to 2 mg/kg/day), 18% (*n* = 10) had a concomitant prescription of po ibuprofen (20 mg/kg/8 h), and 50% (*n* = 27) had a concomitant prescription of inhaled nitrous oxide (6 to 9 L/mn, 15 mn session, three times per day maximum). We proposed different possibilities to each child for evaluating their own pain: VAS (using a little ruler), NPRS (with numbers from 0 to 10), or FPS (with faces expressing pain). Most of the children aged more than 8 years preferred the NPRS, but we observed a good consistency between the different methods for the nociceptive part of the pain. The mean score of the pain scale (VAS, NPRS, or FPS-R) at Day 2 was 6/10 (Q1:5; Q3:7). A total of 44 patients (81%) were still hospitalized four days after arrival. For these 44 patients, at Day 4, the average morphine intake was still high (1.2 mg/kg/d (Q1:0.8; Q3:1.5)). The mean score of the pain scale (VAS, NPRS or FPS-R) at Day 4 was 5/10 (Q1:3; Q3:7). These data are detailed in Table 1.

### 3.3. Prevalence and Description of Neuropathic Pain during the Vaso-Occlusive Crisis

**Figure 1:**
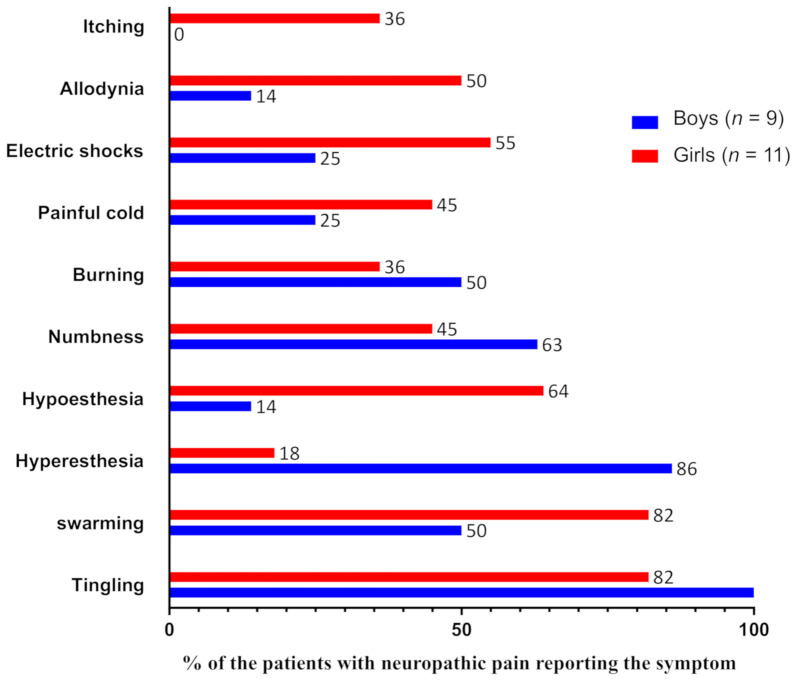
Characteristics of neuropathic symptoms depending on gender.

On Day 2, 20 patients (37%) had a positive DN4 score, indicating the occurrence of an early neuropathic component in the VOC. On Day 4, four of these patients had returned home and could not be reassessed. A total of 16 had a second assessment, highlighting that nine (56%) still had positive DN4 scores on Day 4, while neuropathic pain had disappeared for seven (44%) of them. Among the 34 patients with a negative DN4 score on Day 2, 28 of them had a second evaluation on Day 4. A total of 26 (59%) had a negative DN4 score, confirming the absence of NP, while a neuropathic component of the pain had appeared belatedly for two of them (4.5%) with a positive DN4 score (Figure 1). Overall, 41% of the patients (*n* = 22) experienced neuropathic pain during the VOC, usually at an early stage (91% on Day 2). Among these patients, the symptom most often evoked by the children was the sensation of tingling (89%), but also the sensations of swarming (68%), of numbness (53%), of hypoesthesia (44%) or hyperesthesia (44%), of burning (42%), of electric shocks (42%), of painful cold (37%), of allodynia (35%), and, finally, of itching (21%) (Figure 1).

### 3.4. Predictive Factor for Neuropathic Pain during VOC

We looked more closely at the characteristics of patients with neuropathic pain in order to understand whether there might be risk factors for developing NP. Among the 22 children experiencing NP at least one time during hospitalization, 59% were girls, the median age was 14 years old (Q1:10; Q3:16), and 59% (*n* = 13) were considered as a severe phenotype of SCD because they had more than three hospitalized VOCs in the last 12 months and/or a past history of acute chest syndrome. A total of 73% had chronic treatment with hydroxyurea. The VOCs affected limbs in 73% of cases, with a median pain scale (VAS, NPRS, or FPS-R) of 6 (Q1:5; Q3:8) at Day 2 and 5 (Q1:4; Q3:7) at Day 4 in this population. The median opioid intake in this group was 1.3 mg/kg/day (Q1:1.0; Q3:1.7) at Day 2 and 1.3 mg/kg/day (Q1:0.9; Q3:1.6) at Day 4. In the group without NP during the VOCs, none of the characteristics described above were statistically different from one group to the other (Table 1).

## 4. Discussion

The primary approach to the management of acute VOCs is pain control. The key element in the management of VOCs is the evaluation of pain (its location and intensity, but also its different components) in order to propose an analgesia tailored to the patient, his/her painful experience, and the crisis he/she is going through. Acetaminophen (paracetamol) and Non-Steroids Anti-Inflammatory Drugs (NSAIDs) are widely used adjuvant therapies with opioids [15,16]. Potential adjuvant treatments include intravenous hyperhydration, anxiolytics, local lidocaine patches, and less common non-pharmacologic approaches, such as hypnosis, relaxation, local hot water bottles, a transcutaneous electrical nerve stimulator (TENS), massage, acupuncture, yoga, and meditation [17]. Neuropathic pain is a direct consequence of a lesion or disease affecting the somatosensory system [18]. In SCD, neuropathic lesions may occur rapidly during VOC due to inflammation and acute ischemic/reperfusion injury [19,20], and are supposed to be resolved a few days after the end of the crisis [21]. However, these descriptions most often concern adult patients, and few are reported in children, mainly due to diagnostic difficulties. Indeed, the diagnosis of neuropathic pain during VOC raises the problem of fine descriptions of the characteristics of the pain, which are sometimes difficult in young children, especially during an intensely painful episode where many sensations are mixed, including anxiety, fear of parental separation, and adverse effects of treatment (pain related to infusion devices, opioid-induced pruritus or hyperalgesia, etc.). Moreover, the diagnosis between “pure neuropathic” and “mixed pain” in children and adolescents is very difficult for several reasons: We do not dispose of many diagnostic tools, as questionnaires validated for adults and the electrophysiologic methods, such as quantitative sensory testing (QST) or electromyography (EMG), cannot be easily used in children [22,23]. In a recent survey study among members of learned societies or groups whose members are known to treat pediatric pain, de Leeuw et al. [11] observed that only 45.3% of the respondents used the IASP criteria for establishing the diagnosis of neuropathic pain in children, while 18 (15.4%) explicitly answered that they did not use these criteria; 46 persons (39.3%) left this question unanswered. Most practitioners base the diagnosis of neuropathic pain on a combination of physical examination, history, and/or underlying disease. Otherwise, the underlying pathologies are often different from those encountered in adults. Conditions such as postherpetic neuralgia, radiculopathies, and complications of stroke are very rare in young patients. However, some neuropathic conditions can be recognized in children and adolescents, including phantom limb pain, spinal cord injury, trauma, postoperative neuropathic pain, and the consequences of cancer disease processes and treatment. There are also neuropathic pain syndromes that are rare and specific to the pediatric population (toxic and metabolic neuropathies, hereditary neurodegenerative disorders, mitochondrial disorders). In all cases, it is important to consider the possibility of neuropathic pain or a neuropathic component in mixed pain, since the treatment is quite different from that for nociceptive or inflammatory pain. Severe and long-lasting pain problems in childhood might result in persistence of pain or development of other chronic pain states in adulthood. In the case of VOC, the mechanisms of pain are certainly mixed, including vascular occlusion and the consequent inflammation, leading to mast cell activation, neurogenic inflammation, peripheral nociceptor sensitization, and then to central sensitization and modulation of neural circuits in the brain [4].

The use of the DN4 scale in the form of a picture frame allowed us to take the time to help children describe their pain and characterize its components as well as possible. Thus, we were able to show, for the first time, the high prevalence of neuropathic pain (41% of the patients in our study) during acute VOC in SCD children. This neuropathic component would have gone completely unrecognized and, therefore, under-estimated and under-treated if it had not been researched with adapted tools. We have focused on looking for specific clinical profiles or factors favoring the onset of neuropathic pain in these children. However, the comparison of the groups with and without neuropathic pain showed no statistically different values of phenotypic profiles (no more severe children in the NP group than in the non-NP group), sex ratio, median age, or pain location, contrary to other studies that found a higher prevalence of pain in SCD women and adolescents [24,25]. We also did not find any differences in terms of Hydrocyurea (HU) treatment between these two groups, contrary to the data reported by Brandow et al. [24]. In this study, patients on HU treatment were also older, which may constitute an interpretation bias. However, the effect of these factors on NP cannot be completely ruled out; the size of our sample may have limited the statistical power of the tests for demonstrating differences. One can wonder if the initiation of treatments could change the assessment of neuropathic pain in these children and thus introduce a bias in our study. We would answer to this question that there was no significant difference between the subjects of our cohort concerning the analgesic treatments initiated at the beginning of their hospitalization; most of the children received an intravenous infusion of morphine, Néfopam, paracetamol, and sometimes non-steroidal anti-inflammatory drugs. There were no treatments of neuropathic pain prescribed between Day 2 and Day 4. Thus, if there is a treatment bias, it is homogeneous across the entire cohort. Considering the opioid consumption, we also compared the two groups. Indeed, if hyperalgesia is a signature of neuropathic pain, it may also be caused by prolonged opioid use. Increasing the dose of opioids for pain management may paradoxically increase pain sensitivity, lead to higher pain scores, and be accompanied by allodynia [26]. Nevertheless, in our study, patients with NP did not have higher doses of opioids than children without NP, partially excluding the diagnosis of opioid-induced hyperalgesia. The other treatment that could have an impact on NP is the inhaled nitrous oxide. Indeed, nitrous oxide is not described as a treatment for neuropathic pain per se, but it does have an action on NMDA receptors [27] and, therefore, is susceptible to action on the central sensitization mechanisms. Its usefulness in the management of VOC has been demonstrated to be analgesic and anxiolytic [28], but could also have interesting complementary effects—notably, anti-neuropathic effects—which remain to be studied in the context of VOC. The effectiveness of drugs usually used for neuropathic pain, including gabapentin, pregabalin, amitriptyline, serotonin-noradrenaline reuptake inhibitors (SNRIs), and tricyclic antidepressants, has not been firmly established in SCD patients, let alone in SCD children. Another prospective study may be useful to determine if specific treatment against NP in these patients could reduce the consumption of opioid treatments and/or the length of the hospital stay.

One of the limitations of this study is the lack of data concerning the persistence of these pains until the end of the crisis, or even beyond. In our study, we reported acute neuropathic pain, which sometimes appears to be transient given that 44% of the patients with NP at Day 2 had no more NP at Day 4. Nevertheless, we did not evaluate the persistence or the recurrence of NP after the resolution of the VOC. It is possible that some of the neuropathic pain may persist after the VOC or even become chronic in some patients, requiring long-term treatment.

## 5. Conclusions

Our study shows that neuropathic pain is very common during VOC in SCD children and needs to be specifically screened and treated. The absence of identified risk factors should prompt us to be vigilant regardless of the patient’s age, sex, and clinical presentation. A better understanding and screening of this painful component would lead to a more efficient management of crises. An evaluation of the efficiency and impact of specific treatments for neuropathic pain seems to be essential in SCD children.

## Figures and Tables

**Table 1:** Demographic data and description of the vaso-occlusive crises (Visual Analog Scale (VAS), Consumption of morphine, and DN4 scale results)

	All Patients (*n* = 54)	Patients with NP * (*n* = 22)	Patients without NP ** (*n* = 32)	*p*-Value
**Age Median (Q1; Q3)**	15.0 (11.0; 16.0)	14.0 (10.0; 16.0)	15.0 (13.0; 16.5)	0.11
**Sex Ratio F/M**	1.7	1.4	1.9	0.63
**Genotype**	SC: 6% (*n* = 3)	SC: 14% (*n* = 3)	SC: 0%	-
	SS: 83% (*n* = 45)	SS: 77% (*n* = 17)	SS: 88% (*n* = 28)	
	Sbêta°: 11% (*n* = 6)	Sbêta°: 9% (*n* = 2)	Sbêta°: 12% (*n* = 4)	
**Hydroxyurea treatment**	78% (*n* = 42)	73% (*n* = 16)	81% (*n* = 26)	0.517
Severe SCD phenotype ***	68.5% (*n* = 37)	59% (*n* = 13)	75% (*n* = 24)	0.246
**Characteristics of the episode**				
**Limb pain**	69% (*n* = 37)	73% (*n* = 16)	66% (*n* = 21)	0.58
**VAS at Day 2 Median (Q1; Q3)**	6.0 (5.0; 7.0)	6.0 (5.0; 8.0)	6.0 (4.5; 7.0)	0.72
**Consumption of Morphine at Day 2 Median (Q1; Q3)**	1.2 (0,9; 1,6)	1.3 (1.0; 1.7)	1.1 (0.9; 1.5)	0.52
**DN4 scale ≥ 4 at Day 2**	37% (*n* = 20/54)	91% (*n* = 20/22)	0	-
**VAS at Day Median (Q1; Q3)**	5.0 (3.0; 7.0)	5.0 (4.0; 7.0)	5.0 (3.0; 7.0)	0.45
**Consumption of Morphine at Day 4 Median (Q1; Q3)**	1.2 (0.8; 1.5)	1.3 (0.9; 1.6)	1.1 (0.7; 1.5)	0.49
**DN4 scale ≥ 4 at Day 4**	25% (*n* = 11/44)	50% (*n* = 11/22)	0	-

* Patients were considered to have neuropathic pain (NP) if they had a DN4 score ≥ 4 on Day 2 and/or on Day 4 of the hospitalization. ** Patients were considered to be without NP if they had a DN4 score < 4 on Day 2 and on Day 4 of the hospitalization. *** Severe phenotype was defined by three or more hospitalized VOCs in the last 12 months and/or past history of Acute Chest Syndrome (ACS). F/M: Female /Male.
